# Nonuniqueness of hydrodynamic dispersion revealed using fast 4D synchrotron x-ray imaging

**DOI:** 10.1126/sciadv.abj0960

**Published:** 2021-12-22

**Authors:** Yongqiang Chen, Holger Steeb, Hamidreza Erfani, Nikolaos K. Karadimitriou, Monika S. Walczak, Matthias Ruf, Dongwon Lee, Senyou An, Sharul Hasan, Thomas Connolley, Nghia T. Vo, Vahid Niasar

**Affiliations:** 1 Department of Chemical Engineering and Analytical Science, The University of Manchester, Manchester M13 9PL, UK.; 2 Institute of Applied Mechanics (CE), University of Stuttgart, Stuttgart, Germany.; 3 School of Chemical and Energy Engineering, Universiti Teknologi Malaysia, 81310 Johor Bahru, Johor, Malaysia.; 4 Diamond Light Source Ltd., Diamond House, Harwell Science and Innovation Campus, Didcot, Oxfordshire, OX11 0DE, UK.

## Abstract

Experimental and field studies reported a significant discrepancy between the cleanup and contamination time scales, while its cause is not yet addressed. Using high-resolution fast synchrotron x-ray computed tomography, we characterized the solute transport in a fully saturated sand packing for both contamination and cleanup processes at similar hydrodynamic conditions. The discrepancy in the time scales has been demonstrated by the nonuniqueness of hydrodynamic dispersion coefficient versus injection rate (Péclet number). Observations show that in the mixed advection-diffusion regime, the hydrodynamic dispersion coefficient of cleanup is significantly larger than that of the contamination process. This nonuniqueness has been attributed to the concentration-dependent diffusion coefficient during the cocurrent and countercurrent advection and diffusion, present in contamination and cleanup processes. The new findings enhance our fundamental understanding of transport processes and improve our capability to estimate the transport time scales of chemicals or pollution in geological and engineering systems.

## Teaser

4D x-ray imaging of transport in porous media shows that hydrodynamic dispersion is larger during cleanup versus contamination.

## INTRODUCTION

The characterization of solute transport is important to many subsurface processes, such as contaminant hydrogeology (*1*) and soil remediation (*2*, *3*), seawater intrusion into coastal aquifers (*4*), leachate of fertilizer nutrients in agricultural systems (*5*), as well as in engineering applications (*6*, *7*), such as enhanced oil recovery (*8*). There are two key processes in the named applications: increase in the resident chemical concentration as a result of injection of chemicals at higher concentrations compared to the initial resident concentration referred to as loading (e.g., saltwater intrusion) and decrease in the resident chemical concentration as a result of injection of chemicals at lower concentrations compared to the initial resident concentration referred to as unloading (e.g., soil cleanup or low-salinity waterflooding). There are studies in the literature showing longer unloading process compared to the loading process at the same injection rate. For example, De Smedt and Wierenga (*9*) showed that at the same injection rates, the unloading process required 1.4 times longer time compared to the loading process in an unsaturated glass bead packing [see figure 5 in (*9*)]. Similarly, Huang *et al.* (*10*) reported 2.5 times longer time for the unloading versus loading process in a 12.5-m-long, horizontal heterogeneous soil column. In a recent two-dimensional (2D) micromodel study, similar observations were reported (*11*) demonstrating larger hydrodynamic dispersion coefficient during the unloading compared to the loading. All former experimental studies show that even for nonreactive solutes under the same hydrodynamic conditions, the unloading process is much slower than the loading one. However, no clear and physically based explanation for the effect of transport direction (loading versus unloading) on the transport time scale has been provided. Different transport time scales mean different hydrodynamic dispersion coefficients during loading and unloading for the same hydrodynamic conditions, while in numerous analytical and numerical studies, an identical hydrodynamic dispersion coefficient has been applied to both processes, and nonunique hydrodynamic dispersion coefficient as a function of flow dynamics has not been accounted for (*12*, *13*).

Almost all reported 3D experimental measurements are point measurements, either with a probe inside or at the boundary of porous media with very little information (almost none) about the resident concentration field in the porous media. However, there are very few exceptions. Recently, 4D, high-resolution, synchrotron-based x-ray computed tomography (sXRCT) has been used to delineate the transport process under unsaturated (commonly referred to as two-phase flow) conditions in a glass bead packing (*14*). In addition, with the use of optical imaging (*15*) and the magnetic resonance imaging method (*16*–*18*), hydrodynamic dispersion and velocity field were studied in 3D glass bead packing. Here, we used fast, 4D, synchrotron x-ray imaging of single-phase flow experiments to provide two critical contributions that can lead to revisiting the theory of transport in geosystems. (i) The relation between the hydrodynamic dispersion coefficient and the pore velocity for loading and unloading is established and the nonunique behavior is explained, and (ii) a valuable dataset comprising 4D resident concentration fields at different injection rates for loading and unloading is provided.

## RESULTS

### Dynamic solute distribution during loading and unloading

**Fig. 1.**
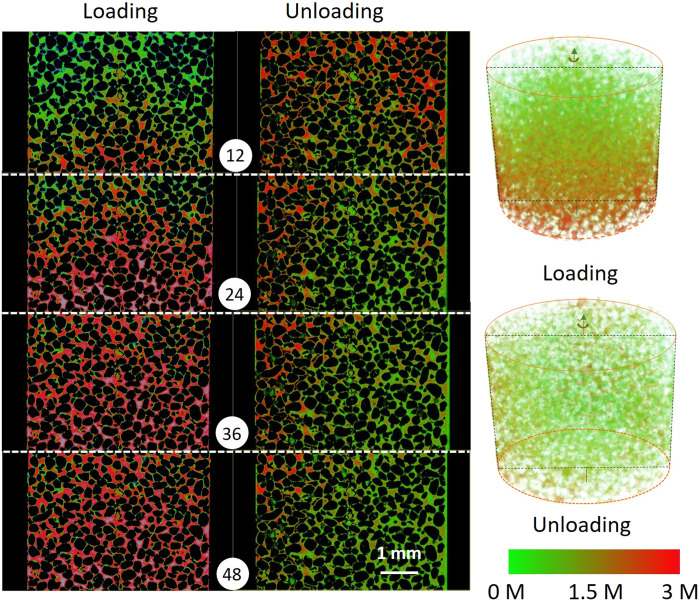
2D cross-sectional presentation of concentration field along the flow direction at an injection rate of 1.6 μl/s. The flow direction is from bottom to top, and the numbers in circles indicate time in seconds. The red to green color indicates the KI concentration in water from high (3 M) to low (0 M) concentrations. The right panel shows the 3D presentation of the concentration field for the same experiments.

The fast 4D x-ray imaging of solute transport generated a valuable dataset and the possibility to visualize the evolution of full concentration field at the pore scale. In Fig. 1, the 2D cross sections of the sand packing and the corresponding concentration field at different times, for loading and unloading, at the rate of 1.6 μl/s, respectively, are presented. To demonstrate the ratio of advection to diffusion, we used the pore-scale Péclet number. The pore-scale Péclet number is defined as $\mathrm{Pe}=\frac{\mathrm{vL}}{D}$, where *v* denotes the pore velocity, *L* is the characteristic length (pore size), and *D* is the diffusion coefficient. The peak pore body diameter (refer to fig. S2) is 90 μm, and the diffusion coefficient for KI in water is equal to 2.44 × 10^−9^ m^2^/s (*19*). Using the pore velocity (estimated from the injection rate divided by the effective cross-sectional area), the Péclet number for the injection rate of 1.6 μl/s is 21.75, which does not correspond to a highly advective transport (*20*) but representative for many natural subsurface systems such as aquifers. In Fig. 1, the flow direction is upward, meaning from bottom to top. Red and green colors indicate high and low KI concentrations, respectively. During the loading process, we observed a relatively homogeneous global and local KI distribution. As shown in Fig. 1 (loading column), a parabolic shape of the KI concentration front is visible. This indicates a faster flow at the center of the sample compared to the sides of the sample. This was possible due to side wall (no flow) boundary conditions. In addition, note that the entrance effect (*17*) may lead to these concentration profiles too, which was also observed in the micromodel experiments (*21*). However, given that the inlet covered the whole cross section of the flow cell, the entrance effect was not expected to be considerable in these experiments.

Figure 1 (unloading column) shows four snapshots of the 2D concentration field during unloading for 12, 24, 36, and 48 s. Unlike the loading process, the KI concentration front during unloading is rather irregular. For example, in the left section of the frame at 24 s, a high KI concentration remains in the sample, while the deionized water front reached the outlet of the field of view. The irregularity in the concentration distribution during the unloading was not related to the velocity field, as the sample packing and injection rates and boundary conditions were exactly identical to those during the loading process. Thus, comparing the concentration fields for loading versus unloading, we can hypothesize that the difference in the concentration fields is exclusively related to the interaction between advection and diffusion. This will be further investigated in the follow-up section.

### Temporal evolution of average resident concentration

**Fig. 2.**
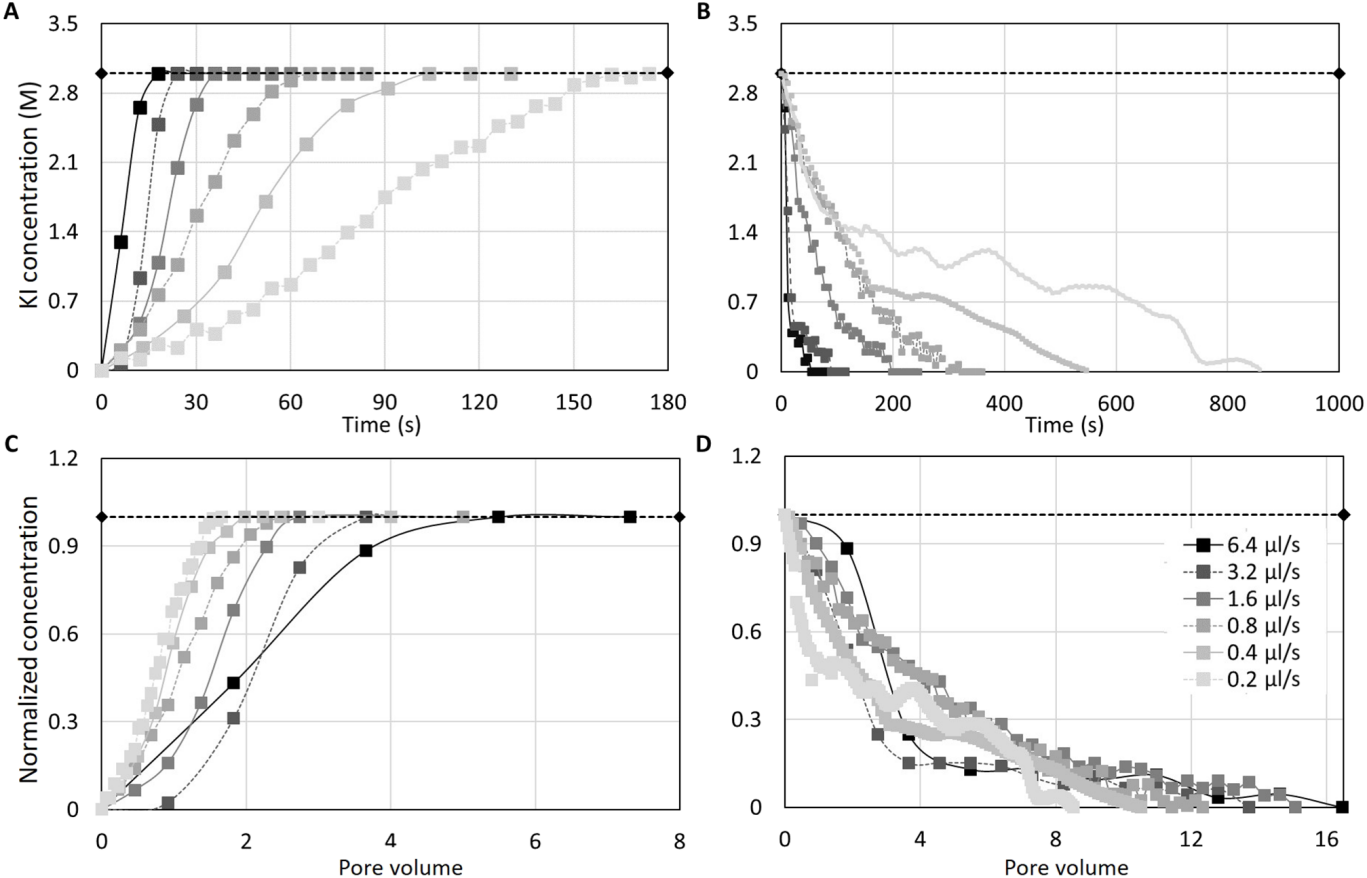
Variation of the resident concentration within the field of view with time and pore volume, quantified on the basis of the sXRCT images. (**A**) The loading experiments at six injection rates. (**B**) The unloading experiments at six injection rates. The normalized average resident concentration (with respect to 3 M concentration) versus the injected pore volumes are shown for the loading (**C**) and unloading (**D**), respectively. The horizontal dash lines denote a reference resident concentration of 3.0 M.

To address this hypothesis, we performed experiments at different injection rates corresponding to different Péclet numbers. The injection rates varied between 0.2 and 6.4 μl/s, corresponding to Péclet numbers varying between 2.7 and 87, respectively. The average resident concentrations versus time were estimated (Fig. 2). The experimental data show that even without macroscopic heterogeneity, the unloading process was around four to five times slower than the loading one for the same injection rate. Note that these results have been established on the basis of the averaging of the resident pore-scale concentration. For example, for the injection rate of 6.4 μl/s, the average resident KI concentration decreased from 3 to 0 M within 54 s, whereas the average resident KI concentration changed from 0 to 3 M during the loading process in 18 s. Just to establish a comparison basis, we estimated the times required to reach 50% of the final concentration for the loading cases ($t_{50}^{l}$) an d the time to reach 50% of the initial concentration for the unloading cases ($t_{50}^{\text{ul}}$). The corresponding values for $t_{50}^{l}$ were 9, 12, 42, 84, 102, and 150 s for injection rates of 6.4, 3.2, 1.6, 0.8, 0.4, and 0.2 μl/s, respectively. Similarly, values of $t_{50}^{\text{ul}}$ were 45, 78, 156, 240, 450, and 762 s for the same injection rates as in the loading case, respectively. This leads to a delay ratio ($t_{50}^{\text{ul}}/t_{50}^{l}$) of 5, 6.5, 3.7, 2.85, 4.4, and 5.08 for the rates of 6.4, 3.2, 1.6, 0.8, 0.4, and 0.2 μl/s, respectively.

The average residual concentration during the loading process shows a smooth behavior. However, for lower fluxes such as 0.2 μl/s (*Pe* = 2.7), in which diffusion is the dominant transport mechanism, there were some fluctuations in the concentration curves. This might be due to the redistribution of concentration inside the field of view. However, it is not possible to assess the cause of the fluctuations for this specific case. To confirm that there was no instability effect due to large density contrast or centrifugal effect due to the rotation of the sample for imaging, we performed further analysis as reported in the Supplementary Materials. We can conclude that that the effect of density-dependent instability, viscosity contrast effect, and centrifugal force are negligible in the reported experiments.

The unloading process, especially for slow injection fluxes, showed long tailing. As an example, the average resident concentration decreased quickly from 3 to 1 M in 150 s for the injection rate of 0.4 μl/s. However, the dilution of the remaining 1 M required additional 396 s. This tailing is characteristic of a non-Fickian behavior, which is mostly pronounced at high rates where two distinct transport time scale (e.g., advective versus diffusive) of transport were observed (*22*–*24*). The presence of stagnant regions was even reported in fully saturated porous materials (*25*). However, the pore-scale x-ray images of this study did not show detectable stagnant regions. Thus, we do not expect considerable stagnant regions in this fully saturated homogeneous sand packing. Thus, the long tailing cannot be related to the presence of a fully stagnant (diffusion-controlled) region. To compare the experiments based on a dimensionless time, we converted time to injected pore volumes using the following equation *Q* × *t*/*V*_p_, in which *Q* and *V*_p_ denote the volumetric flux and the pore volume, respectively. Normalized concentration curves versus the injected pore volumes are shown in Fig. 2 (C and D). Figure 2D shows that for higher fluxes during unloading, a tailing of the concentration is visible, which reflects the non-Fickian behavior. However, such a non-Fickian behavior is not visible during the loading process. This observation again implies the nonunique behavior of transport during the corresponding processes for the same injection rate and the same porous medium.

**Fig. 3.**
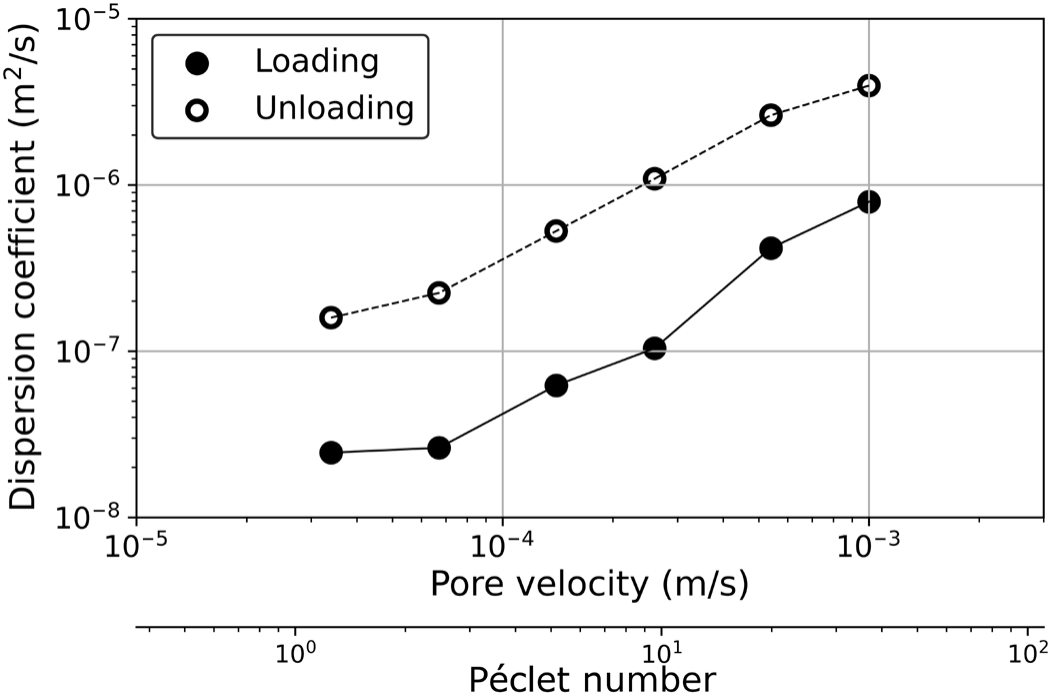
Nonuniqueness of hydrodynamic dispersion coefficient for loading and unloading experiments at the same injection rates. The figure shows the hydrodynamic dispersion coefficient versus the pore velocity and the corresponding Péclet number for loading and unloading processes. Hydrodynamic dispersion coefficient and pore velocity were estimated by fitting the advection-dispersion equation as explained in the Supplementary Materials.

To explicitly illustrate this nonuniqueness, we estimated the corresponding hydrodynamic dispersion coefficients for each process and injection rate by fitting an analytical advection-dispersion equation. Note that the fitting results report the hydrodynamic dispersion as the summation of diffusion and mechanical dispersion. Details of the fitting procedure can be found in the Supplementary Materials. The results for the hydrodynamic dispersion coefficient versus the pore velocity (and equivalent Péclet number) are shown in Fig. 3. The hydrodynamic dispersion coefficient for loading (*D*^l^) is smaller than the hydrodynamic dispersion for the unloading process (*D*^ul^). The difference between *D*^l^ and *D*^ul^ can be larger than one order of magnitude for some cases. However, the difference slightly decreases with the decrease in the injection flux (i.e., Péclet number). This again emphasized the fact that difference in values of hydrodynamic dispersion is induced by the competition between the advection and diffusion transport.

## DISCUSSION

### New knowledge about the hydrodynamic dispersion

These results highlight the critical gaps and potential misconceptions in the application of hydrodynamic dispersion for practical problems: (i) A nonunique relation between the hydrodynamic dispersion coefficient and pore velocity has been identified for loading versus unloading, even for such a small sand packing, which has not been addressed in the literature before. (ii) With a decrease in the Péclet number, the difference between *D*^l^ and *D*^ul^ becomes smaller.

**Fig. 4.**
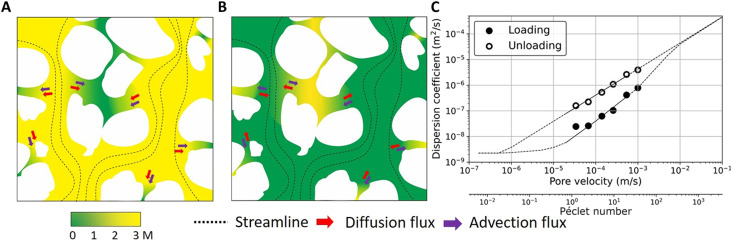
Proposed transport diagram delineating the nonuniqueness of hydrodynamic dispersion coefficient as a function of Péclet number. Schematic pore-scale presentation of cocurrent and countercurrent advective and diffusive transport mechanisms during loading (**A**) and unloading (**B**), respectively. Proposed transport diagram (**C**), which suggests for large and very small Péclet numbers, the discrepancies between the loading and unloading hydrodynamic dispersion coefficient reduce.

Hydrodynamic dispersion is, by definition, the summation of molecular diffusion and mechanical dispersion, and this is what is being shown in Fig. 4C. For large Péclet numbers, mechanical dispersion is the dominant part, so the two processes, loading and unloading, should exhibit similar behaviors. Assuming that there is no spatial concentration heterogeneity, the same should stand for the case of very low Péclet number, where molecular diffusion is the dominant process. For intermediate Péclet numbers though, it is expected that the advective forces will create a heterogeneous concentration field, strongly affecting the corresponding molecular diffusion, depending on the process followed.

On the basis of this trend, it can be conjectured that for a mixed advection-diffusion regime, the countercurrent and cocurrent advection and diffusion transport leads to the observed difference, and with an increase in the Péclet number, the nonlinear interaction between diffusion and advection decreases. To be specific, the advection and diffusion act in the same direction during the loading process (as shown in Fig. 4). However, given that diffusion is governed by the chemical potential gradient, it acts in the opposite direction of advection during unloading (Fig. 4). We conjecture that because of a nonlinear interaction between these two transport processes, a significant difference between the time scales of loading and unloading processes was resulted. A potential reason for the nonlinearity can be the concentration-dependent diffusion coefficient, which has been formerly addressed in the literature (*26*) but was considered negligible in the hydrodynamic dispersion for porous media applications. Dunlop and Stokes (*19*), Carey *et al.* (*27*), and other literature (*19*, *27*–*29*) showed that concentration-dependent diffusion coefficients indicate a decreasing diffusion coefficient with the increase in KI concentration. We performed a molecular dynamics simulation, and a similar trend was verified (refer to the Supplementary Materials). Given the spatial distribution of concentration field during loading and unloading, the diffusion transport during loading and unloading will not be spatially similar. However, the heterogeneous concentration field is highly controlled by the advective flux. This leads us to the hypothesis that for a range of Péclet numbers, a nonunique hydrodynamic dispersion versus Péclet number for loading versus unloading is expected. These results and explanations led us to propose a new transport diagram (Fig. 4C). Because, with increasing Péclet number, the difference between *D*^ul^ and *D*^l^ decreases, it is justifiable to assume that a unique hydrodynamic dispersion coefficient would be obtained for high Péclet numbers (≫100), as in this case, diffusion is negligible. In addition, for no-flow conditions, transport will be purely controlled by a unique diffusion coefficient. On the basis of the results and physical-based justifications, we propose a transport diagram that has not been addressed in the literature. Note that the range of pore velocity and pore-scale Péclet number in many transport phenomena in geological systems such as aquifers are within the loop presented in Fig. 4C (*30*). The result is of significant importance for better estimation of the time scale of transport processes especially the unloading processes related to contaminant hydrogeology, soil remediation, and groundwater quality modeling.

## MATERIALS AND METHODS

### Materials and fluids

Deionized water and a 3 M KI water solution were used for the loading and unloading experiments. The porous medium was a sand packing made of acid-washed sand grains sieved using a 150-μm sieve size. The sample diameter was 4.5 mm, and the imaged height was 3.47 mm with a porosity of 37.8% and pore volume of 21 μl. To derive the pore size distribution, we extracted the pore network from the XRCT images using the maximal ball algorithm (refer to the Supplementary Materials) (*31*). The extracted pore network had 6452 pore bodies and 24,205 throats. The histogram (with 100 bins) and the cumulative probability distribution of pore radii are shown in fig. S2. There are two peaks in the pore size distribution histogram, representing the pore throat and pore body radii with 10 and 45 μm, respectively. An intrinsic permeability of 4.8 × 10^−12^ m^2^ was estimated from flow field simulations.

### Experimental setup and imaging

The key feature of the experiments and this study is the reconstruction of the in situ concentration field based on sXRCT that provided the microscale, spatiotemporal information of transport for different injection fluxes. To quantify the transient variation of solute concentration, we performed 4D fast sXRCT during transport processes. We used monochromatic sXRCT at the Diamond Light Source, beamline I12 (*32*) to establish the correlation between the concentration and CT number (referred to as the calibration curves) and achieve high-resolution images in time (3 s of scanning and 3 s of data acquisition time) and space (3.25 μm) with minimum artifacts. Following the methodology established by Hasan *et al.* (*24*), the reference time (*t* = 0) was established on the basis of the change in the x-ray intensity value for the field of view. The calibration curve established for the sXRCT values versus the actual KI solution concentration for the range of 0.1 to 3 M did not show any sensitivity of the errors to the concentration. Given the similar sensitivity of XRCT values at high and low concentrations for the loading and unloading, within the selected range of KI, start and end times were determined.

For each scanning frame, the sample was projected 600 times with the photon energy of 53 keV, with each projection lasting for 0.005 s. The raw data were reconstructed using I12 in-house Python codes (*33*). A data-processing pipeline includes a flat-field correction; zinger removal (*33*), which is a process for removing the image artifact in the form of a bright straight or zinger; ring artifact removal (*34*); denoising by a low-pass filter (*33*); automated determination of the center of rotation (*35*); and reconstruction using a direct Fourier inversion method (*36*, *37*). The reconstructed data were then prepared for segmentation and statistical analysis. Note that during the imaging of one full scan (3 s), the concentration field would evolve too. Thus, the reconstructed image is the integration of all the projections (0.005 s per projection) of the scanning period (3 s). After 600 projections, the collected data would be averaged to get the responding x-ray intensity at each location of the sample. The fluid distribution was assumed not to change markedly in this short projection time for the slow flow rates. However, because of this technical limitation, it was not possible to image the transport of solutes at injection rates higher than 6.4 μl/s. Nevertheless, yet at high flow rate (such as 6.4 μl/s), x-ray imaging can still capture the dynamic evolution of KI concentration field.

The experimental setup is schematically shown in fig. S1. The flow cell was made of polyether ether ketone with dimensions of 4.8 mm in diameter and 50 mm in height, filled with fine sand grains. The flow cell was connected to two syringe pumps for the injection of deionized water and the KI solution. There was a back pressure of 0.5 bar to avoid the gas bubble generation during the exposure to high-energy x-ray radiation. The sample was positioned in the vertical direction and injection was from the bottom of the sample. To prepare the clean water-saturated sample, the sample was saturated with deionized water at a high injection rate of 10 μl/s. Samples saturated with the KI solution were initiated at the same rate.

Each experiment was continued at excessive time to accommodate hundreds of pore volume. After the initialization of the samples, either loading or unloading was performed: injection of 3 M KI solution at a controlled rate into the sample filled with deionized water (referred to as loading) or injection of deionized water at a controlled rate into the sand packing filled with 3 M KI solution (referred to as unloading). The loading and unloading experiments were performed at rates of 0.2, 0.4, 0.8, 1.6, 3.2, and 6.4 μl/s. During the whole injection, x-ray images were taken with 600 projections for each tomography, and, in total, more than 23 terabytes of data were produced and analyzed for imaging a field of view with a diameter of 4.5 mm and a height of 3.47 mm.

## Supplementary Materials

### This PDF file includes:

Supplementary Methods Figs. S1 to S6 References
