# Supplementary material for: Nonuniqueness of hydrodynamic dispersion revealed using fast 4D synchrotron x-ray imaging
Source: Sci Adv. 2021 Dec 22;7(52):eabj0960. doi: 10.1126/sciadv.abj0960 (PMC8694585; doi:10.1126/sciadv.abj0960)
Supplement: Supplementary file 1 — Supplementary Methods Figs. S1 to S6 References [file sciadv.abj0960_sm.pdf]

Science Advances
AAAS

Supplementary Materials for

Nonuniqueness of hydrodynamic dispersion revealed using fast 4D synchrotron x-ray imaging

Yongqiang Chen, Holger Steeb, Hamidreza Erfani, Nikolaos K. Karadimitriou, Monika S. Walczak, Matthias Ruf, Dongwon Lee, Senyou An, Sharul Hasan, Thomas Connolley, Nghia T. Vo, Vahid Niasar*

*Corresponding author. Email: vahid.niasar@manchester.ac.uk

Published 22 December 2021, *Sci. Adv.* **7**, eabj0960 (2021)
DOI: 10.1126/sciadv.abj0960

This PDF file includes:

Supplementary Methods
Figs. S1 to S6
References

This supplementary information provides detailed information about the experimental setup, image processing method and upscaling the transport properties.

Methods

Experimental setup

The experimental setup is schematically shown in Fig.S1. We employed synchrotron X-ray Computed Tomography (sXRCT) and captured the 3D concentration field in the porous domain every 6 seconds, at the spatial resolution of 3.25 μm/pixel. The flow cell was a cylinder with a length of 50 mm, and by 4.8 mm in diameter, filled with fine sand grains with the mean size of 150 μm. The fluids used were water and a KI-water solution at 3 mol/l concentration, serving as a tracer for imaging transport within the water phase. KI solution was used as a contrast agent. The high X-ray attenuation coefficient of iodine relative to water increases X-ray absorption and provides better attenuation contrast for determining the presence of the fluid in which the KI is dissolved. All experiments were performed under flow-controlled conditions, and a back pressure of 0.5 Bar to avoid bubble generation during the high-energy X-ray imaging.

Synchrotron X-ray Computed Tomography (sXRCT) images analysis of the solid phase

Using the maximal ball method (*31*), the pore size distribution for the whole network was extracted. The histogram and cumulative probability of pore radii is shown in Fig. S2. The distribution is bimodal, as the smaller peak corresponds to the pore throat radii and the larger peak corresponds to the pore body radii.

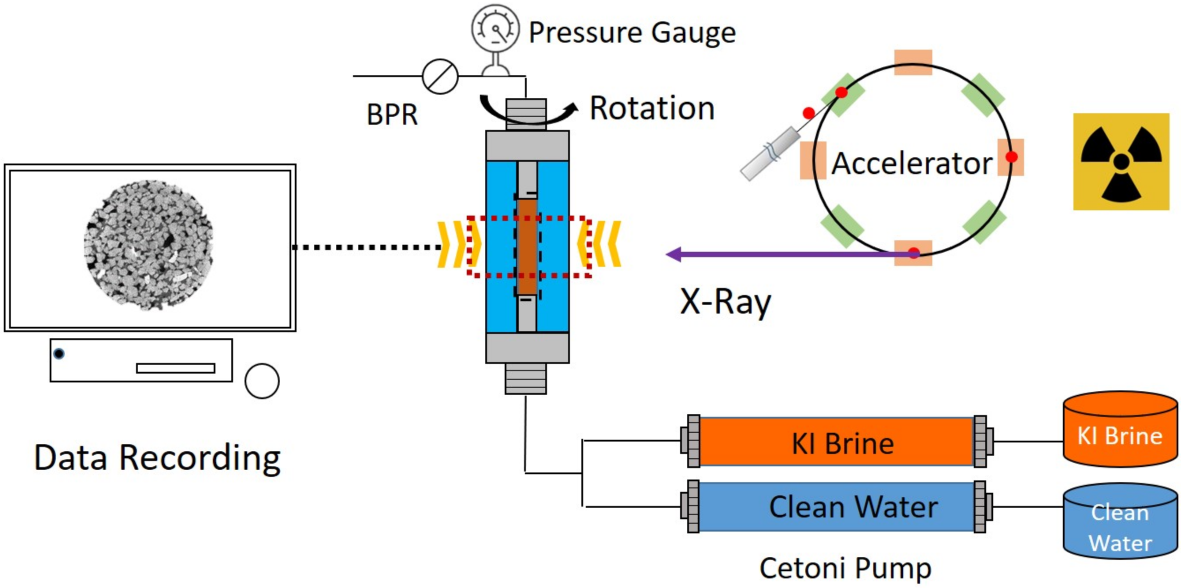

Figure S1: **Scheme of the solute transport experimental setup and X-ray imaging.** Note that the dimensions of the experimental apparatus are not scaled with actual size. The injection direction to the sample is from bottom to the top. The pairs of loading and unloading experiments were performed at the same injection rates for 6 difference rates ranging between 0.2 to 6.4 μl/s. The holder was filled with fine sand and the fluids used were 3M potassium iodide (KI)-water solution and de-ionized water.

X-ray image analysis of the concentration field

The sXRCT images were processed with Fiji (*38*) and Avizo with the assistance of the OpenCV library (*39*). First, the collected 32-bit images were transformed to 16-bit images in Fiji. Then, the images of water-saturated samples were selected to make a mask of the pore space. The pore space was partitioned out with the use of the watershed algorithm in Avizo. Uaing the OpenCV library, the binary images were masked on grey scale images to remove the solid phase. The masked images were processed in Fiji with the "analyze particles" function to get the mean CT intensity of the pore space.

To establish a relation between CT values and actual concentrations, four calibration experiments were performed. Four solutions with known KI concentrations (0.1, 1, 2, 3 mol/l) were

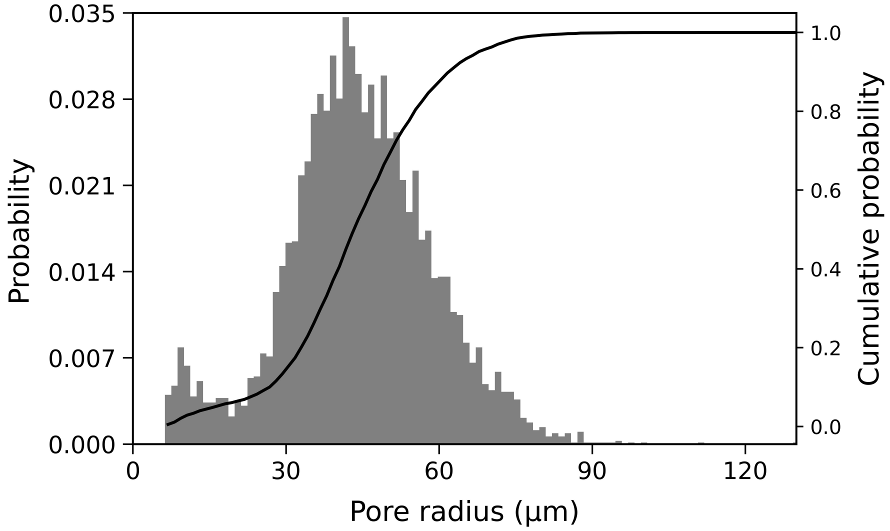

Figure S2: **Histogram and cumulative probability distribution of pore radii.** Pore size distribution was extracted using image processing. Left y-axis shows the probability of each bin size and the right axis shows the cumulative probability.

injected into the sandpacking and the CT values were obtained. Due to the nearly linear CT response with the actual KI concentration (as shown in Fig. S3), the solute concentrations were then computed based on Eq. 1 (*40, 41*). We did not apply any filters to the grey scale images in order to keep the original CT intensities rather than lose information during the filtration process,

$$C_{voxel} = \frac{I_{voxel} - I_{water}}{I_{3\,mol/l} - I_{water}} \times 3\text{ mol/L} \tag{1}$$

where C_{voxel} refers to the solute concentration in the voxel of interest, I_{voxel}, $I_{3\,mol/l}$ and I_{water} indicate the grey scale value of the same voxel, 3 mol/l KI and clean water, respectively.

Estimation of upscaled hydrodynamic dispersion coefficient and pore velocity

To obtain the pore velocity and hydrodynamic dispersion coefficients during loading and unloading, we fitted the average resident concentration curves to the analytical advection-dispersion

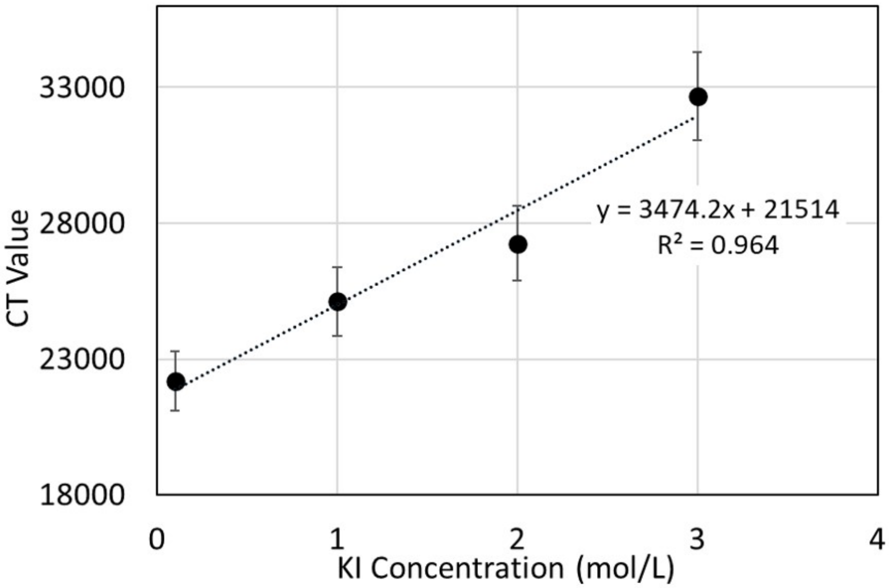

Figure S3: **sXRCT response at different KI concentrations referred to as the calibration experiments**. This relation was established from the calibration experiments at concentrations ranging between 0.1M to 3M with 3 to 5 repetition per concentration. The results demonstrate a linear relation between the sXRCT values and the physical concentration of the solution. The error bars do not show a correlated variability with the concentration.

equation. Given that the field of view is away from the inlet boundary, concentration at the boundary of the field of view was not equal to the reservoir concentration at time equal to 0. Due to the time-dependence of the inlet concentration, an analytical equation (Eqs. 5-10) with the time dependent concentration at the inlet boundary (Eq. 3) and a finite domain (Eq. 4) was employed. The equation has been presented as case A11 in the reference (*42*).

Initial and boundary conditions:

$$C\left(x,0\right)=C_i \tag{2}$$

$$C\left(0,t\right)=C_a+C_b\times^{-\lambda t} \tag{3}$$

$$\frac{\partial C\left(L,t\right)}{\partial t}=0 \tag{4}$$

Analytical Solution:

$$C\left(L,t\right)=C_i+\left(C_a-C_i\right)A\left(x,t\right)+C_bB\left(x,t\right) \tag{5}$$

where

$$\begin{aligned}A\left(x,t\right)=&\frac{1}{2}\text{erfc}\left[\frac{Rx-vt}{2\sqrt{DRt}}\right]+\frac{1}{2}\exp\left(\frac{vx}{D}\right)\text{erfc}\left[\frac{Rx+vt}{2\sqrt{DRt}}\right]\\&+\frac{1}{2}\left[2+\frac{v\left(2L-x\right)}{D}+\frac{v^2t}{DR}\right]\exp\left(\frac{vL}{D}\right)\text{erfc}\left[\frac{R\left(2L-x\right)+vt}{2\sqrt{DRt}}\right]\\&-\sqrt{\frac{v^2t}{\pi DR}}\exp\left[\frac{vL}{D}-\frac{R}{4Dt}\left(2L-x+\frac{vt}{R}\right)^2\right]\end{aligned} \tag{6}$$

$$B\left(x,t\right)=\exp\left(-\lambda t\right)\frac{B_3\left(x,t\right)}{B_4\left(x\right)} \tag{7}$$

where

$$\begin{aligned}B_3\left(x,t\right)=&\frac{1}{2}\exp\left[\frac{\left(v-y\right)x}{2D}\right]\text{erfc}\left[\frac{Rx-yt}{2\sqrt{DRt}}\right]\\&+\frac{1}{2}\exp\left[\frac{\left(v+y\right)x}{2D}\right]\text{erfc}\left[\frac{Rx+yt}{2\sqrt{DRt}}\right]\\&+\frac{y-v}{2\left(y+v\right)}\exp\left[\frac{\left(v+y\right)x-2yL}{2D}\right]\text{erfc}\left[\frac{R\left(2L-x\right)-yt}{2\sqrt{DRt}}\right]\\&+\frac{y+v}{2\left(y-v\right)}\exp\left[\frac{\left(v-y\right)x+2yL}{2D}\right]\text{erfc}\left[\frac{R\left(2L-x\right)+yt}{2\sqrt{DRt}}\right]\\&+\frac{v^2}{2\lambda DR}\exp\left[\frac{vL}{D}+\lambda t\right]\text{erfc}\left[\frac{R\left(2L-x\right)+vt}{2\sqrt{DRt}}\right]\end{aligned} \tag{8}$$

$$B_4\left(x\right)=1+\frac{y-v}{y+v}\exp\left(\frac{-yL}{D}\right) \tag{9}$$

and

$$y = v\sqrt{1 - \frac{4\lambda DR}{v^2}} \tag{10}$$

For the loading experiments $C_i = 0$, $C_a = 1$. For the unloading experiments, $C_i = 1$, $C_a = 0$. In all experiments $R = 1$ was fixed. A least square method was used to guarantee the best fitting of hydrodynamic dispersion coefficients (as shown in Fig. S4). Rate of concentration evolution at the inlet boundary (λ [s^{-1}]) is proportional to v^2/D with a constant coefficient for all cases. For each injection rate of loading and unloading, as the hydrodynamic conditions should have been identical. Thus, the pore velocity was directly estimated knowing the injection rate, porosity and permeability of the sample. Hydrodynamic dispersion coefficients for loading (D^l), and unloading (D^{ul}) processes were fitted separately.

Impact of Rayleigh-Taylor instability and centrifugal forces on solute transport

Rayleigh instability due to density contrast

Due to significant density contrast between the 3 mol/l KI solution and water ($\Delta\rho = 400 kg/m^3$), it is important to estimate the density-dependent instability during the unloading process (i.e., injection of the lighter fluid from the bottom). Rayleigh instability analysis applied for the miscible fluids in porous media defines the Rayleigh number under "no flow" conditions. Note that in the experiments of this study the Ra number is expected to be smaller, due to the upwards direction of viscous forces, which hamper the downward forces due to the density difference. Thus, the Rayleigh instability will be even further suppressed in these injections scenarios. Following (*43, 44*), Ra should be smaller than the critical Rayleigh number (Ra_c) to have the

stability in transport:

$$Ra = \frac{\Delta\,\rho\,g\,K\,H}{D\,\mu} < Ra_c = 4\,\pi^2, \tag{11}$$

$$\frac{400\,\mathrm{Kg/m^3} \times 9.8\,\mathrm{m/s^2} \times 4.8 \times 10^{-12}\,\mathrm{m^2} \times 3.47 \times 10^{-3}\,\mathrm{m}}{2.5 \times 10^{-9}\,\mathrm{m^2/s} \times 1 \times 10^{-3}\,\mathrm{Kg/(m \cdot s)}} = 26.1 < 39.4, \tag{12}$$

, where $\Delta\rho$ is the density difference between two fluids, K is the intrinsic permeability of the porous medium, H is the height, g is the gravitational acceleration, ϕ is the porosity, D is the diffusion coefficient, and μ is water viscosity. Ra =26.1 is smaller than the critical Rayleigh number (*43, 44*). Thus, we can conclude that that role of density-dependent instability in our observations was negligible.

Effect of the Centrifuge force

To calculate the centrifuge force and its importance in the data analysis, the centrifuge force was calculated at the outmost part of the flow cell.

Since the sample rotated π radians in 3 seconds, the angular velocity (ω) was 1.05 radians per second. Given that the outmost radius of the sample was r = 2.25mm, the centrifugal force acting on a mass in a pore with the diameter of 50μm would be : $F_r \approx \omega^2 r \rho D^3 = 4.13 \times 10^{-13} N$. For the same control volume using the Hagen-Poisseuille flow, the viscous force for the smallest flow rate would be approximated as $F_v = 8Q\mu l/R^2 = 1.7 \times 10^{-11} N$. As shown, even for the smallest flow rate the centrifugal force will be negligible. Additionally, the ratio of centrifuge to the gravity acceleration is $\bar{F} = \omega^2 r/g = 1.05^2 \times 2.25 \times 10^{-3}/9.8 = 2.5 \times 10^{-4}$. This also indicates negligible centrifuge acceleration compared to the gravity.

Diffusion coefficients from molecular dynamics simulation

To assess the change of diffusion coefficients with concentration, we calculated the diffusion coefficients by molecular dynamics simulation in different KI concentration solutions at 0.01

mol/L, 0.1 mol/L, and 1 mol/L. The molecular dynamic simulation was taken in LAMMPS (*45*). The simulation input were prepared with assistance of Moltemplate (*46*). The SPC/E water (*47*) was used and a force field for KI solvent was from Koneshan et al. (*48*). The non-bonded potential between different molecules was modelled with arithmetic mixing method. The simulation boxes are shown in Fig. S5. In box A and C, it was initially filled with water molecules while box B was filled with KI solution. The simulation includes two steps. At step one, the three boxes were relaxed in NVE ensemble with a Langevin thermostat at 300K under periodic boundary conditions in x and y directions while a fixed boundary condition was in z condition. A wall was applied in z direction to avoid atoms lost. The three simulations were run for 25 ns to fully relax the system. After the system was relaxed, the three boxes were combined together and ran in a NVT ensemble under periodic boundary conditions in three directions. The dynamic diffusion coefficients were calculated with Einstein equation (Equ. 13) as shown in Fig. S5.

$$\lim_{t\to\infty} \langle |r(t) - r(0)|^2 \rangle = 6Dt \tag{13}$$

Where $r(t)$ is the position of molecule at time t, D is diffusion coefficient, t is time, $\langle\rangle$ means the ensemble average.

Impact of concentration-dependent diffusion coefficient on transport time scale

To demonstrate qualitatively the impact of concentration-dependent diffusion coefficient on transport time scale, we performed 1D numerical simulations using the conventional advection-diffusion equation. $\partial C/\partial t = -v\partial C/\partial x + D\partial^2 C/\partial x^2$. Fig. S5 and literature work (Matuura et al. (*28*),Harned et al. (*29*), Dunlop et al. (*19*), and Carey et al. (*27*)) showed that diffusion coefficients decrease with increase of the concentration. Since the exact parameters for this specific

KI concentration range is not available, we assumed as simple relation as $D = D_m(1 - \alpha C)$. For a 1D system with the length of 5×10^{-3}m, velocity of 10^{-5}m/s and $D_m = 2.5 \times 10^{-9}$m^2/s, simulations for the loading and unloading scenarios were done (Fig. S6). To demonstrate the difference between loading and unloading due to concentration-dependent diffusion, we defined the deviation factor as $C_{load} - 1 + C_{unload}$ shown in Fig. S6b, which show positive values. The average resident concentration profiles for loading and unloading scenarios are shown in Fig. S6c. Clearly, our results show that even for a 1D case (fixed velocity) the effect of concentration-dependent diffusion coefficient is visible. Note that this mechanisms will be more pronounced in high-concentration gradients such as saline aquifers (*27*). Additionally, for a 3D system with a large variation of pore velocity, it is expected that delay in unloading scenarios to be more pronounced than the small systems.

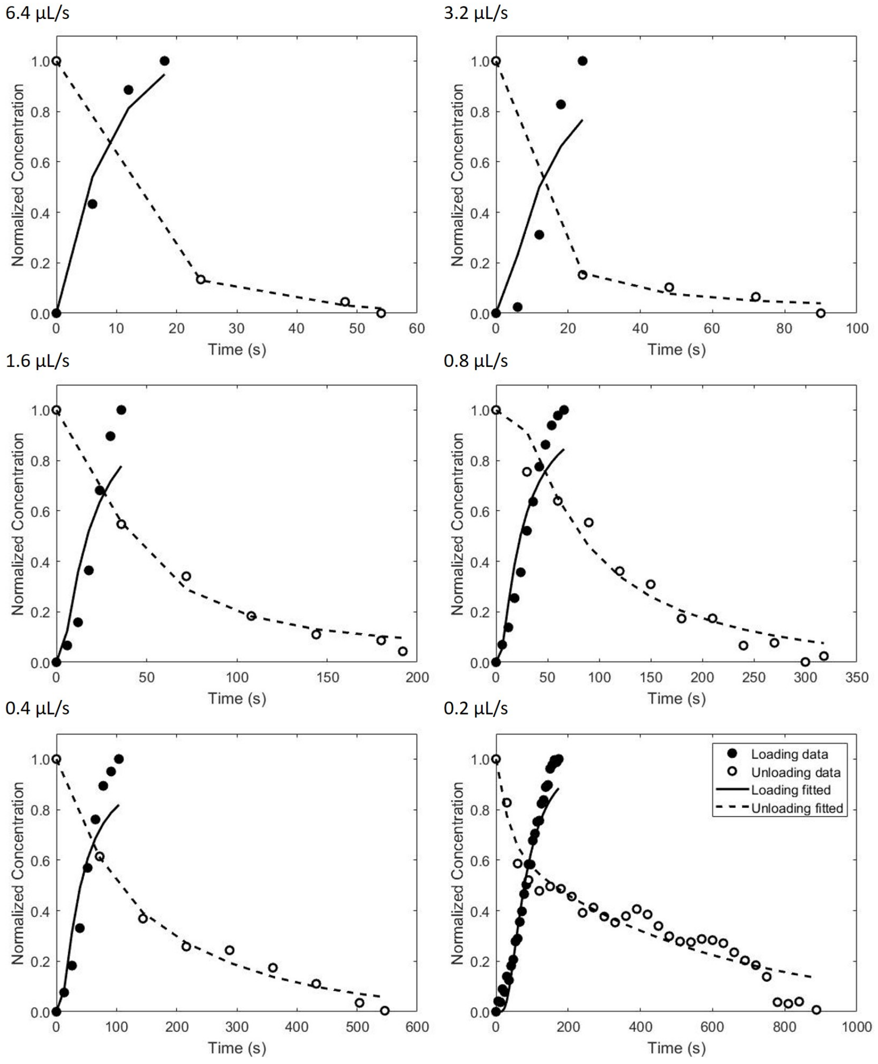

Figure S4: **The fitting results using Eqs 2-10 versus the experimental measurements for the resident concentration in the field of view**. Loading and unloading experiments were fitted simultaneously.

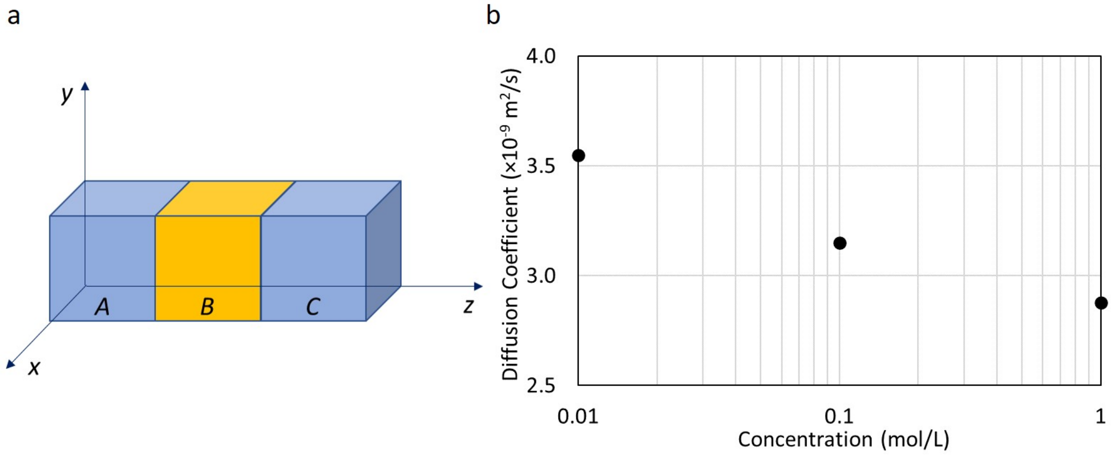

Figure S5: **Decrease of the diffusion coefficient with increase of KI concentration ranging from 0.1M to 1M, resulted from the molecular dynamics simulations.**(a) schematic graph of molecular dynamics simulation boxes; (b) calculated diffusion coefficients of I^- vs KI concentration.

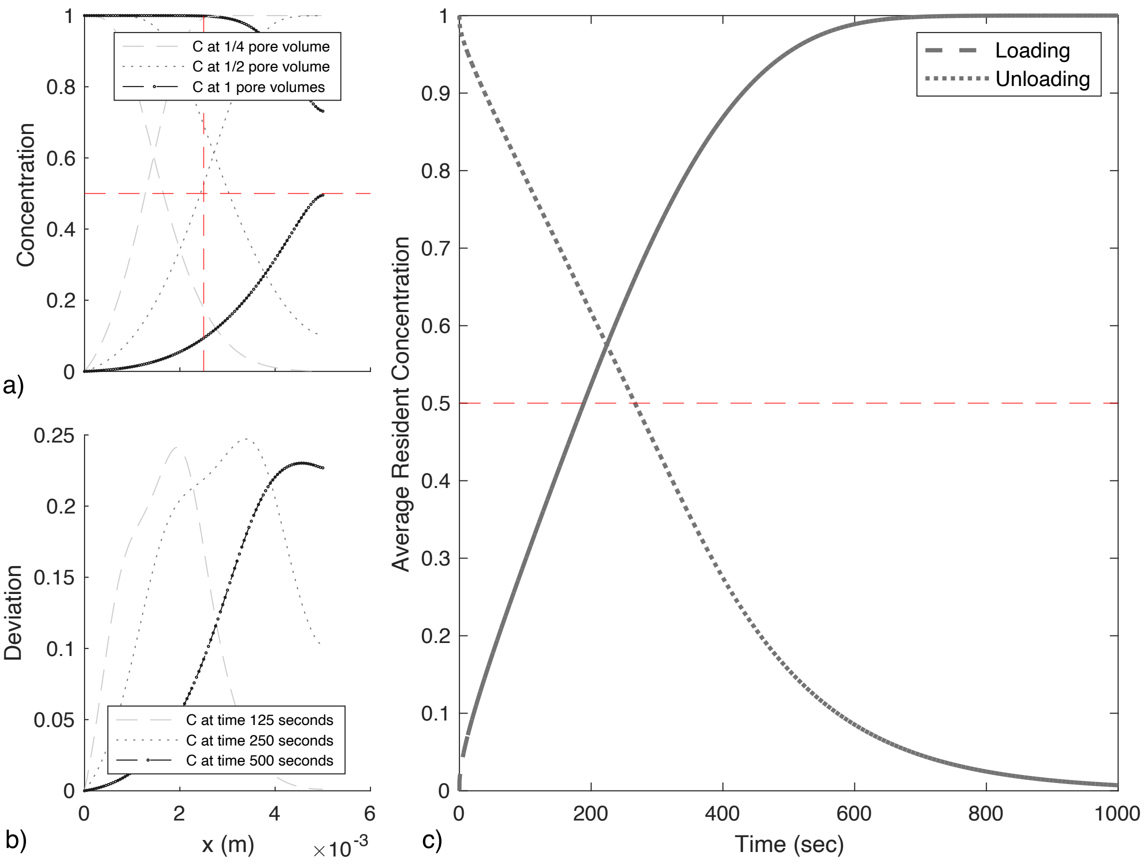

Figure S6: **1D simulation of advection-diffusion simulation for loading and unloading scenarios with concentration-dependent diffusion.** a) Concentration profiles along distance for loading and unloading at 0.25, 0.5 and 1 pore volume injection. b) Deviation between the loading and unloading concentrations at a given distance defined as $C_{load} - 1 + C_{unload}$. The positive values of deviation show that loading is faster than unloading. c) Average resident concentration for loading and unloading scenarios.

REFERENCES AND NOTES

1. M. J. Ascott, D. C. Gooddy, L. Wang, M. E. Stuart, M. A. Lewis, R. S. Ward, A. M. Binley, Global patterns of nitrate storage in the vadose zone. *Nat. Commun.* **8**, 1416 (2017).

2. R. Kumar, F. Heße, P. Rao, A. Musolff, J. Jawitz, F. Sarrazin, L. Samaniego, J. Fleckenstein, O. Rakovec, S. Thober, S. Attinger, Strong hydroclimatic controls on vulnerability to subsurface nitrate contamination across europe. *Nat. Commun.* **11**, 6302 (2020).

3. V. Joekar-Niasar, B. Ataie-Ashtiani, Assessment of nitrate contamination in unsaturated zone of urban areas: The case study of tehran, iran, *Environ. Geol.* **57**, 1785–1798 (2009).

4. A. D. Werner, C. T. Simmons, Impact of sea-level rise on sea water intrusion in coastal aquifers. *Groundwater* **47**, 197–204 (2009).

5. L. Harper, R. Sharpe, G. Langdale, J. Giddens, Nitrogen cycling in a wheat crop: Soil, plant, and aerial nitrogen transport. *Agron. J.* **79**, 965–973 (1987).

6. G. F. Froment, K. B. Bischoff, J. De Wilde, *Chemical Reactor Analysis and Design* (Wiley New York, 1990), vol. 2.

7. O. Levenspiel, Chemical reaction engineering. *Ind. Eng. Chem. Res.* **38**, 4140–4143 (1999).

8. R. Aziz, V. Niasar, H. Erfani, P. J. Martínez-Ferrer, Impact of pore morphology on two-phase flow dynamics under wettability alteration. *Fuel* **268**, 117315 (2020).

9. F. De Smedt, P. Wierenga, Solute transfer through columns of glass beads. *Water Resour. Res.* **20**, 225–232 (1984).

10. K. Huang, N. Toride, M. T. Van Genuchten, Experimental investigation of solute transport in large, homogeneous and heterogeneous, saturated soil columns, *Transp. Porous Media* **18**, 283–302 (1995).

11. H. Erfani Gahrooei, N. Karadimitriou, A. Nissan, M. Walczak, S. An, B. Berkowitz, V. Niasar, Process-dependent solute transport in porous media. *Transp. Porous Media* **140**, 421–435 (2021).

12. A. Polak, A. S. Grader, R. Wallach, R. Nativ, Chemical diffusion between a fracture and the surrounding matrix: Measurement by computed tomography and modeling. *Water Resour. Res.* **39**, 1106 (2003).

13. R. M. Greenwald, S. M. Gorelick, Particle travel times of contaminants incorporated into a planning model for groundwater plume capture. *J. Hydrol.* **107**, 73–98 (1989).

14. S. Hasan, V. Niasar, N. K. Karadimitriou, J. R. A. Godinho, N. T. Vo, S. An, A. Rabbani, H. Steeb, Direct characterization of solute transport in unsaturated porous media using fast x-ray synchrotron microtomography. *Proc. Natl. Acad. Sci. U.S.A.* **117**, 23443–23449 (2020).

15. M. Souzy, H. Lhuissier, Y. Méheust, T. Le Borgne, B. Metzger, Velocity distributions, dispersion and stretching in three-dimensional porous media. *J. Fluid Mech.* **891**, A16 (2020).

16. M. Deurer, I. Vogeler, B. E. Clothier, D. R. Scotter, Magnetic resonance imaging of hydrodynamic

dispersion in a saturated porous medium. *Transp. Porous Media* **54**, 145–166 (2004).

17. A. Lehoux, S. Rodts, P. Faure, E. Michel, D. Courtier-Murias, P. Coussot, Magnetic resonance imaging measurements evidence weak dispersion in homogeneous porous media. *Phys. Rev. E* **94**, 053107 (2016).

18. J. Raimbault, P.-E. Peyneau, D. Courtier-Murias, T. Bigot, J. Gil Roca, B. Béchet, L. Lassabatère, Investigating the impact of exit effects on solute transport in macroporous media. *Hydrol. Earth Syst. Sci.* **25**, 671–683 (2021).

19. P. Dunlop, R. Stokes, The diffusion coefficients of sodium and potassium iodides in aqueous solution at 25°[1]. *J. Am. Chem. Soc.* **73**, 5456–5457 (1951).

20. M. Babaei, V. Joekar-Niasar, A transport phase diagram for pore-level correlated porous media. *Adv. Water Resour.* **92**, 23–29 (2016).

21. N. K. Karadimitriou, V. Joekar-Niasar, M. Babaei, C. A. Shore, Critical role of the immobile zone in non-fickian two-phase transport: A new paradigm. *Environ. Sci. Technol.* **50**, 4384–4392 (2016).

22. N. K. Karadimitriou, V. Joekar-Niasar, O. G. Brizuela, Hydro-dynamic solute transport under two-phase flow conditions. *Sci. Rep.* **7**, 6624 (2017).

23. S. An, S. Hasan, H. Erfani, M. Babaei, V. Niasar, Unravelling effects of the pore-size correlation length on the two-phase flow and solute transport properties: Gpu-based pore-network modeling. *Water Resour. Res.* **56**, e2020WR027403 (2020).

24. S. Hasan, V. Joekar-Niasar, N. K. Karadimitriou, M. Sahimi, Saturation dependence of non-fickian transport in porous media. *Water Resour. Res.* **55**, 1153–1166 (2019).

25. K. Coats, B. Smith, Dead-end pore volume and dispersion in porous media. *Soc. Pet. Eng. J.* **4**, 73–84 (1964).

26. D. W. McCall, D. C. Douglass, The effect of ions on the self-diffusion of water. I. Concentration dependence. *J. Phys. Chem.* **69**, 2001–2011 (1965).

27. A. E. Carey, S. W. Wheatcraft, R. J. Glass, J. P. O'Rourke, Non-fickian ionic diffusion across high-concentration gradients. *Water Resour. Res.* **31**, 2213–2218 (1995).

28. R. Matuura, Y. Koga, Self-diffusion of iodide ion and strontium ion in strontium iodide solutions. *Bull. Chem. Soc. Jpn.* **32**, 1143–1148 (1959).

29. H. S. Harned, R. L. Nuttall, The differential diffusion coefficient of potassium chloride in aqueous solutions. *J. Am. Chem. Soc.* **71**, 1460–1463 (1949).

30. V. Rønde, U. McKnight, A. Sonne, N. Balbarini, J. Devlin, P. Bjerg, Contaminant mass discharge to streams: Comparing direct groundwater velocity measurements and multi-level groundwater sampling with an in-stream approach. *J. Contam. Hydrol.* **206**, 43–54 (2017).

31. A. Q. Raeini, B. Bijeljic, M. J. Blunt, Generalized network modeling: Network extraction as a coarse-scale discretization of the void space of porous media, *Phys. Rev. E* **96**, 013312 (2017).

32. M. Drakopoulos, T. Connolley, C. Reinhard, R. Atwood, O. Magdysyuk, N. Vo, M. Hart, L. Connor, B. Humphreys, G. Howell, S. Davies, T. Hill, G. Wilkin, U. Pedersen, A. Foster, N. D. Maio, M. Basham, F. Yuan, K. Wanelik, I12: The joint engineering, environment and processing (JEEP) beamline at diamond light source. *J. Synchrotron Radiat.* **22**, 828–838 (2015).

33. N. Vo, R. Atwood, M. Drakopoulos, T. Connolley, Data processing methods and data acquisition for samples larger than the field of view in parallel-beam tomography. *Opt. Express* **29**, 17849–17874 (2021.

34. N. T. Vo, R. C. Atwood, M. Drakopoulos, Superior techniques for eliminating ring artifacts in x-ray micro-tomography, *Opt. Express* **26**, 28396–28412 (2018).

35. N. T. Vo, M. Drakopoulos, R. C. Atwood, C. Reinhard, Reliable method for calculating the center of rotation in parallel-beam tomography, *Opt. Express* **22**, 19078–19086 (2014).

36. J. D. O'Sullivan, A fast sinc function gridding algorithm for fourier inversion in computer tomography. *IEEE Trans. Med. Imaging* **4**, 200–207 (1985).

37. D. Gürsoy, F. De Carlo, X. Xiao, C. Jacobsen, Tomopy: A framework for the analysis of synchrotron tomographic data, *J. Synchrotron Radiat.* **21**, 1188–1193 (2014).

38. J. Schindelin, I. Arganda-Carreras, E. Frise, V. Kaynig, M. Longair, T. Pietzsch, S. Preibisch, C. Rueden, S. Saalfeld, B. Schmid, J.-Y. Tinevez, D. J. White, V. Hartenstein, K. Eliceiri, P. Tomancak, A. Cardona, Fiji: An open-source platform for biological-image analysis, *Nat. Methods* **9**, 676–682 (2012).

39. G. Bradski, A. Kaehler, *Learning OpenCV: Computer Vision with the OpenCV library* (O'Reilly Media Inc., 2008).

40. H. M. D. Agbogun, T. A. Al, E. M. A. Hussein, Three dimensional imaging of porosity and tracer concentration distributions in a dolostone sample during diffusion experiments using X-ray micro-ct. *J. Contam. Hydrol.* **145**, 44–53 (2013).

41. Y. Zhang, P. Mostaghimi, A. Fogden, A. Sheppard, A. Arena, J. Middleton, R. T. Armstrong, Time-lapsed visualization and characterization of shale diffusion properties using 4d x-ray microcomputed tomography. *Energy Fuel* **32**, 2889–2900 (2018).

42. M. T. Van Genuchten, *Analytical Solutions of the One-Dimensional Convective-Dispersive Solute Transport Equation* (no. 1661, U.S. Department of Agriculture, Agricultural Research Service, 1982).

43. E. Lindeberg, D. Wessel-Berg, Vertical convection in an aquifer column under a gas cap of CO_2, *Energy Convers. Manag.* **38**, S229–S234 (1997).

44. M. C. K. Kim, Onset of buoyancy-driven convection in a variable viscosity liquid saturated in a porous medium. *Chem. Eng. Sci.* **113**, 77–87 (2014).

45. S. Plimpton, Fast parallel algorithms for short-range molecular dynamics, *J. Comput. Phys.* **117**, 1–19 (1995).

46. A. I. Jewett, D. Stelter, J. Lambert, S. M. Saladi, O. M. Roscioni, M. Ricci, L. Autin, M. Maritan, S. M.

Bashusqeh, T. Keyes, R. T. Dame, J.-E. Shea, G. J. Jensen, D. S. Goodsell, Moltemplate: A tool for coarse-grained modeling of complex biological matter and soft condensed matter physics. *J. Mol. Biol.* **433**, 166841 (2021).

47. H. J. C. Berendsen, J. R. Grigera, T. P. Straatsma, The missing term in effective pair potentials. *J. Phys. Chem.* **91**, 6269–6271 (1987).

48. S. Koneshan, J. C. Rasaiah, R. M. Lynden-Bell, S. H. Lee, Solvent structure, dynamics, and ion mobility in aqueous solutions at 25 °C. *J. Phys. Chem. B.* **102**, 4193–4204 (1998).
